# An Automated knowledge‐based planning routine for stereotactic body radiotherapy of peripheral lung tumors via DCA‐based volumetric modulated arc therapy

**DOI:** 10.1002/acm2.13114

**Published:** 2020-12-03

**Authors:** Justin Visak, Gary Y. Ge, Ronald C. McGarry, Marcus Randall, Damodar Pokhrel

**Affiliations:** ^1^ Medical Physics Graduate Program Department of Radiation Medicine University Kentucky Lexington KY USA

**Keywords:** adaptive re‐planning, FFF‐beam, knowledge‐based planning, lung SBRT

## Abstract

**Purpose:**

To develop a knowledge‐based planning (KBP) routine for stereotactic body radiotherapy (SBRT) of peripherally located early‐stage non‐small‐cell lung cancer (NSCLC) tumors via dynamic conformal arc (DCA)‐based volumetric modulated arc therapy (VMAT) using the commercially available RapidPlan^TM^ software. This proposed technique potentially improves plan quality, reduces complexity, and minimizes interplay effect and small‐field dosimetry errors associated with treatment delivery.

**Methods:**

KBP model was developed and validated using 70 clinically treated high quality non‐coplanar VMAT lung SBRT plans for training and 20 independent plans for validation. All patients were treated with 54 Gy in three treatments. Additionally, a novel *k*‐DCA planning routine was deployed to create plans incorporating historical three‐dimensional‐conformal SBRT planning practices via DCA‐based approach prior to VMAT optimization in an automated planning engine. Conventional KBPs and *k*‐DCA plans were compared with clinically treated plans per RTOG‐0618 requirements for target conformity, tumor dose heterogeneity, intermediate dose fall‐off and organs‐at‐risk (OAR) sparing. Treatment planning time, treatment delivery efficiency, and accuracy were recorded.

**Results:**

KBPs and *k*‐DCA plans were similar or better than clinical plans. Average planning target volume for validation was 22.4 ± 14.1 cc (7.1–62.3 cc). KBPs and *k*‐DCA plans provided similar conformity to clinical plans with average absolute differences of 0.01 and 0.01, respectively. Maximal doses to OAR were lowered in both KBPs and *k*‐DCA plans. KBPs increased monitor units (MU) on average 1316 (*P < *0.001) while *k*‐DCA reduced total MU on average by 1114 (*P < *0.001). This routine can create *k*‐DCA plan in less than 30 min. Independent Monte Carlo calculation demonstrated that *k*‐DCA plans showed better agreement with planned dose distribution.

**Conclusion:**

A *k*‐DCA planning routine was developed in concurrence with a knowledge‐based approach for the treatment of peripherally located lung tumors. This method minimizes plan complexity associated with model‐based KBP techniques and improve plan quality and treatment planning efficiency.

## 1 INTRODUCTION

Stereotactic body radiation therapy (SBRT) of lung tumors is an alternative treatment modality to surgery for early stage non‐small‐cell lung cancer (NSCLC) patients, boasting local control rates greater than 97% at 3‐yr.^1^, ^2^, ^3^ These outstanding clinical outcomes were predominantly based on traditional lung SBRT treatments performed with 7 to 13 coplanar/non‐coplanar three‐dimensional (3D)‐conformal static beams or with a few dynamic conformal arcs (DCA).^4^, ^5^ With modern advances in technology, lung SBRT can be delivered using intensity modulated radiation therapy (IMRT) or volumetric modulated arc therapy (VMAT). VMAT offers the most conformal dose distribution with higher chances of sparing organs‐at‐risk (OAR).^6^ When coupled with a 6 MV flattening filter free (FFF) beam, VMAT benefits are enhanced by providing higher dose rates, reduction in out of field dose, and improved coverage at the lung‐tumor interface when compared to traditionally flattened beams.^7^, ^8^

The generation of a high quality VMAT lung SBRT plan can require multiple iterations of optimization due to difficult patient geometry, tumor size, or location.^9^ In general, inverse planning heavily depends on a planner’s experience, treatment planning time, and planner’s skill. Inter‐planner variability potentially leads to inconsistent plan quality and reduced patient safety.^10^ Efforts have been made to increase treatment planning efficiency and plan quality using a form of inverse planning automation known as knowledge‐based planning (KBP).^11^ Model‐based KBP is a commonly utilized automatic planning strategy that gathers historical treatment planning data to predict achievable OAR doses for a prospective patient.^12^ This form of KBP has demonstrated success in creating dosimetrically similar or better plans when compared to manual planning across many treatment sites with limited but recently increasing literature for lung SBRT.^12^, ^13^, ^14^, ^15^, ^16^, ^17^, ^18^

However, a major concern with using KBP for lung SBRT is its tendency to increase total monitor units (MU) and overall plan complexity^12^, ^18^ which can increase treatment delivery complexity leading to unintended consequences particularly with VMAT plans. This includes the interplay effect between MLC motion and the tumor motion due to breathing cycle.^19^ VMAT lung SBRT for small tumor sizes (<3 cm) could result in small field dosimetry errors.^20^ These drawbacks are compounded by very high MLC modulation observed in knowledge‐based planning. More traditionally planned 3D‐conformal and DCA methods may improve the level of confidence in the treatment. MLC interplay effect and small field dosimetry errors are reduced improving delivery, efficiency, and accuracy in these plans.^19^

Recently, Pokhrel and colleagues have shown that DCA‐based VMAT lung SBRT planning can provide a dosimetrically similar or better plan with reduced complexity when compared to a standard clinical VMAT lung SBRT plans.^21^ Utilizing their approach, in this study we have developed a novel and automated KBP routine using Varian’s RapidPlan^TM^ knowledge‐based planning engine that couples the benefits of a DCA‐based dose technique with modern knowledge‐based VMAT optimization. To deploy this new and clinically useful technique, it was first necessary to develop and validate a robust non‐coplanar VMAT lung SBRT RapidPlan model for medically inoperable/operable early‐stage, peripherally located NSCLC patients. This model was created to fully comply with the RTOG‐0618 lung SBRT protocol’s dosing requirements. Its novelty is furthered because the model uses more accurate advanced Acuros‐based dose calculation for heterogeneity corrections to better predict dose at soft tissue tumor and low‐density lung interfaces.^7^, ^22^, ^23^

## 2 MATERIALS AND METHODS

### 2.A Patient population and clinical plans

Approval from our institutional review board was obtained to utilize 90 clinically treated patients’ treatment plans for peripherally located early stage, NSCLC tumors that met the criteria set forth by RTOG‐0618. Motion management for these patients was primarily performed using abdominal compression unless the patient presented with a comorbidity that did not allow for compression, in these cases a 4D‐CT scan was performed. A gross tumor volume (GTV) was delineated in a lung window and a planning target volume (PTV) was created with added margins of 1.0 cm superior/inferior and 0.5 cm laterally per protocol guidelines. With the 4D‐CT scan, the PTV was generated using a 0.5 cm isotropic margin around the internal target volume (ITV). OARs were contoured per RTOG‐0618 guidelines. Clinical non‐coplanar VMAT plans were created in Varian’s Eclipse treatment planning system (TPS) on a Truebeam Linac (Varian Medical Systems, Palo Alto, CA). Details of patient set up have previously been published elsewhere.^7^ All patients received a total dose of 54 Gy in 3 fractions prescribed to the 70‐80% isodose line.

### 2.B Development and validation of KBP model

The new KBP model was trained and validated using 90 previously treated high quality non‐coplanar VMAT lung SBRT plans. Seventy plans were selected for training and the remaining 20 plans were used for validation. Prior to input, training and validation datasets were manually verified to have correct calculation models and grid sizes (e.g., PO MLC algorithm and Acuros‐XB algorithm enabled). Training contours and overall plan quality were then evaluated for compliance per RTOG‐0618 guidelines. Once the KBP model was verified, normal tissue constraints, and dose objectives were iteratively selected.

To ensure the KBP model was fully functional and robust, 20 independent patients were specifically selected to include both lungs’ geometries, differing tumor locations and variable tumor sizes. This validation dataset included: 6 RUL, 6 LUL, 3 LLL, and 5 RLL patients with an average tumor sizes of 17.0 ± 9.9 cc (7.8–37.5 cc), 19.5 ± 12.1 cc (7.1–42.9 cc), 38.7 ± 18.2 cc (18.0–62.3 cc), and 22.6 ± 10.2 cc (7.8–37.6 cc), respectively. Validation plans were re‐optimized with the RapidPlan model using identical treatment geometry as the clinical plans to create the KBP’s. Plans were normalized to have identical or better target coverage than the original plans. Meaning, they were normalized so that at least 95% of the PTV volume received full prescription dose.

### 2.C Dosimetric comparison criteria

Re‐optimized plans were evaluated for target conformity, dose gradient and intermediate dose spillage as described by RTOG‐0618. Target conformity was assessed using the conformity index defined as the ratio of the 100% isodose line volume to PTV volume. Dose gradient was assessed using the RTOG recommended gradient index (GI) defined as the ratio of the 50% isodose line volume to the PTV volume. The maximum dose 2 cm away from the PTV (D2cm) in any direction and the gradient distance (GD), defined as the average distance between the 100% and 50% isodose lines, were used to quantify degree of intermediate dose fall‐off.

Volumetric and maximum doses to organs at risk outlined by RTOG‐0618 were evaluated. These dose limiting organs (DLO) include the spinal cord, skin, esophagus, trachea, heart, bronchial tree, ribs and normal lung. The total monitor units divided by prescription dose in cGy defined as the modulation factor (MF), including beam‐on time, was used to assess plan complexity. An in‐house data collection method using the Visual Eclipse Scripting Application Programming Interface (ESAPI) (Varian Medical Systems, Palo Alto, CA), Microsoft Excel (Microsoft corp., Redmond, WA), and MATLAB (Math Works, Natick, MA) was developed for rapid collection of the aforementioned data. A paired student t‐test was used to assess statistical agreement (*P < *0.05 statistically significant).

### 2.D A novel *k*‐DCA planning routine

**Fig. 1:**
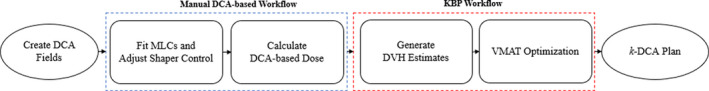
Proposed *k*‐dynamic conformal arcs treatment planning workflow for perepherial lung stereotactic body radiotherapy (SBRT).

To integrate the benefits of both traditional planning techniques and modern lung SBRT treatment practices using VMAT optimization, a routine was developed to improve the plan quality and patient safety in prospective treatments. This routine creates a *k*‐DCA plan using a combination of manual and automated planning approaches with minimal deviation from traditional planning workflow. To begin, planning geometry is manually determined by deploying dynamic conformal arcs and collimator angles. An MLC is then added to each field and is fit with a 2‐mm margin around the PTV on each DCA. Within the PO algorithm (v15.0 or higher) exists the new MLC aperture shaper controller (ACS). Following creation of planning geometry, the ACS is adjusted from its default setting of ‘low’ to ‘very high.’ This is modified to aid in the reduction of MLC modulation in the final plan. Once this aperture setting is applied, a 3D DCA‐based dose is calculated and field weights are adjusted to give a practical starting point and a base dose for the future VMAT optimization. Following the DCA‐based dose calculation, the VMAT optimizer is launched and DVH estimates are automatically generated by enabling the KBP model (see Section 2.B) within the VMAT optimization window. VMAT optimization is performed using the newly and automatically generated dose optimization objectives and priorities via the KBP model. Fig. 1 outlines this process.

### 2.E Independent dose verification

To verify knowledge‐based plans independently, patient‐specific quality assurance was performed using an in‐house Monte Carlo (MC) program.^24^, ^25^ This was performed in lieu of traditional based quality assurance measurements as recent technological advancements in online/offline‐adaptive re‐planning strategies may not allow enough time to perform a conventional physical measurement.^26^ The in‐house MC code uses a vendor provided phase space file to base its functionality off the PENELEOPE MC code.^27^ Rather than physical measurement of multi‐leaf collimators at the machine, a vendor provided schematic was used to model in the MC code and independent dose verification. More details of this algorithm used for this physics second check tool can be found in the cited literature above.

## 3 RESULTS

### 3.A Clinical plans vs KBPs

Knowledge‐based plans produced similar or better target coverage than clinical plans (Table 1). Slight dose escalation to the GTV was achieved with KBPs with an average of 2.2 Gy (*P < *0.001). PTV minimum coverage was maintained while slightly increasing the PTV mean dose (*P < *0.001) with no increase in intermediate dose‐spillage with KBPs. This is reflected in both the lower D2cm and the average reduction of 0.1 cm in the gradient distance (*P < *0.001). For a similar CI, there was significant improvement of GI with KBPs (*P < *0.001) compared to clinical plans indicating less intermediate dose spillage in normal lung (Table 1).

**Table 1:** Evaluation of plan quality and target indices for all 20 lung stereotactic body radiotherapy (SBRT) validation cases generated using knowledge‐based planning (KBP) or k‐dynamic conformal arcs (DCA) routine.

Target	Parameter	Clinical	KBP	*k‐*DCA	Clinical vs KBP	Clinical vs *k*‐DCA	KBP vs *k*‐DCA
GTV	Min (Gy)	58.3 ± 2.1	60.3 ± 2.3	62.0 ± 2.1	***P = *0.002**	***P < *0.001**	***P = *0.002**
	Mean (Gy)	62.4 ± 1.4	64.6 ± 1.9	66.9 ± 1.6	***P = *0.004**	***P < *0.001**	***P < *0.001**
	Max (Gy)	65.5 ± 1.2	67.7 ± 1.5	69.3 ± 1.6	***P = *0.001**	***P < *0.001**	***P < *0.001**
PTV	D99%(Gy)	52.3 ± 0.4	52.5 ± 0.4	52.0 ± 0.6	*P = *n. s.	*P = *n. s.	***P = *0.002**
	Mean (Gy)	59 ± 0.7	61.5 ± 0.8	61.9 ± 0.7	***P* < 0.001**	***P < *0.001**	***P = *0.01**
	CI	1.02 ± 0.03	1.01 ± 0.02	1.03 ± 0.03	*P = *n. s.	*P = *n. s.	***P = *0.001**
	HI	1.21 ± 0.02	1.27 ± 0.02	1.28 ± 0.02	***P < *0.001**	***P < *0.001**	***P < *0.001**
	GI	4.56 ± 0.8	3.95 ± 0.5	4.23 ± 0.6	***P < *0.001**	***P = *0.005**	***P < *0.001**
	D2cm (%)	49.3 ± 5.6	48.6 ± 4.7	50.6 ± 4.6	*P = *n. s.	*P = *n. s.	***P = *0.025**
	GD (cm)	1.04 ± 0.1	0.94 ± 0.1	0.98 ± 0.1	***P < *0.001**	***P = *0.004**	***P = *0.05**

Mean ± SD and *P*‐values were reported. n. s. = not significant. Significant *P*‐values are highlighted in bold. SD = standard deviation.

### 3.B Clinical plans vs *k*‐DCA plans

When the proposed automatic planning routine to create a *k*‐DCA plan was deployed, a higher target dose was achieved at minimal costs to plan quality when compared to clinical plans. The GTV minimum dose was escalated on average 3.7 Gy in *k*‐DCA plans. This is due to the increased average MLC aperture size and less MLC modulation through the target. PTV target metrics showed higher dose with an increase in mean dose by an average of 2.9 Gy (*P < *0.001) with no clinically significant differences in PTV minimum coverage. Despite the higher delivered GTV dose, the CI differences between the *k‐*DCA plans and clinical plans were insignificant. As expected, and following the trend of KBPs, *k*‐DCA plans were more homogenous indicated by the lower GI (*P = *0.005) and delivered less intermediate dose spillage reflected in a lower values of GD (*P = *0.004). D2cm was slightly increased in *k*‐DCA plans with respect to clinical plans but this increase was not statistically significant (Table 1).

### 3.C OAR sparing

**Fig. 2:**
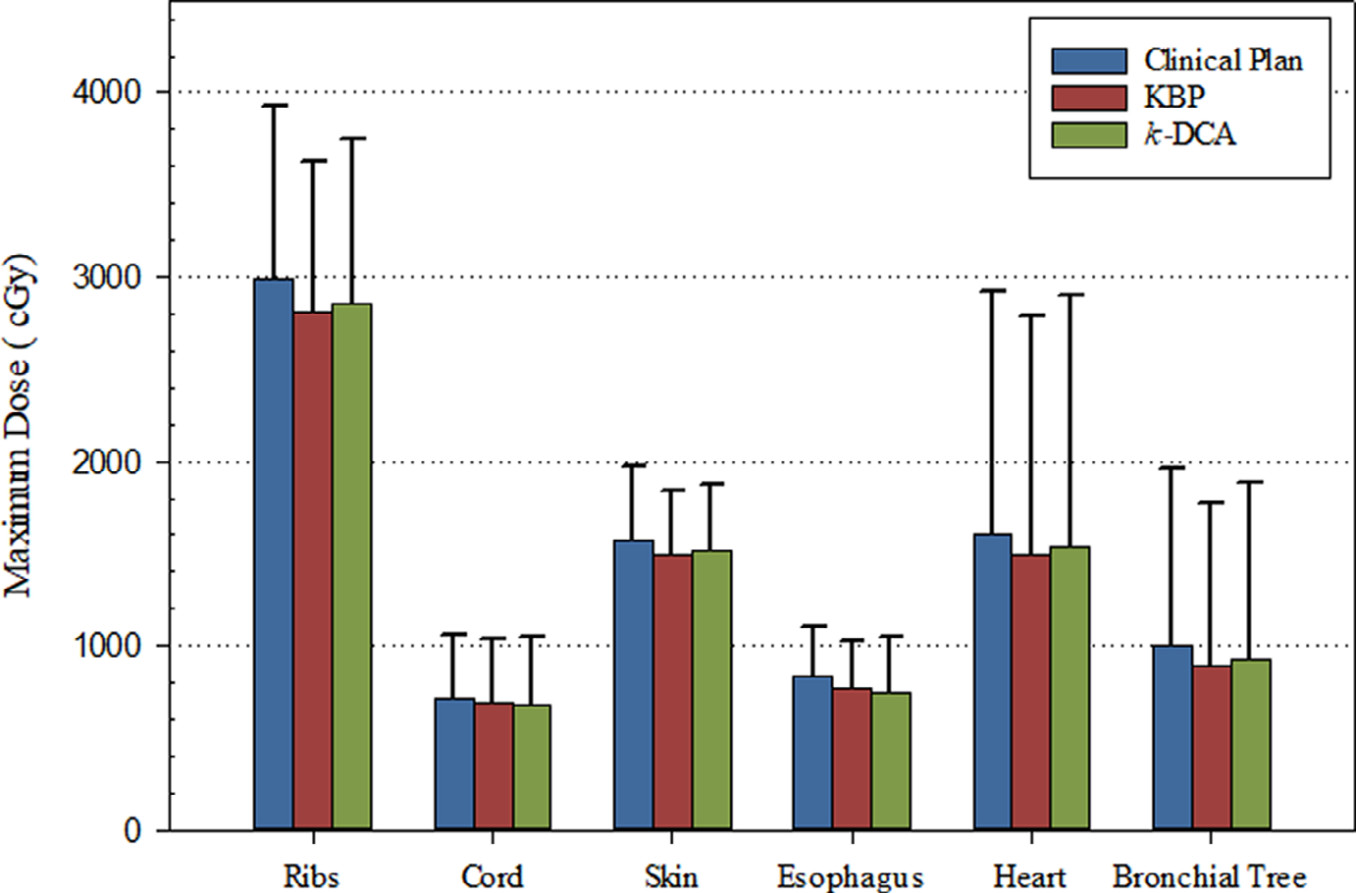
Average maximum doses of selected organs‐at‐risk (OAR) for clinical plans, knowledge‐based plannings (KBPs) and *k*‐dynamic conformal arcs (DCA) plans for all 20 lung stereotactic body radiotherapy validation cases. In all cases both KBPs and *k*‐DCA plans were able to successfully lower the maximum doses delivered to the OAR. KBPs lowered the maximum rib dose by an average of 1.8 Gy and *k‐*DCA reduced the maximum esophageal dose by 0.9 Gy when compared to clinical plans.

All OAR dosing criteria was assessed per RTOG‐0618 protocol’s requirements for all 20 lung SBRT validation cases. Clinically relevant maximal doses to OAR are shown in Fig. 2. Both KBPs and *k‐DCA* plans provided less maximum dose to these select structures. Better OAR sparing can be achieved while still maintaining slight dose escalation to the tumor using the newly developed KBP model or automated *k*‐DCA routine. The maximum ipsilateral brachial plexus doses were not clinically significant (not shown). Volumetric doses were also tracked with notable sparing of 1.0 cc of the ribs in KBPs with average dose reduction of 0.65 ± 1.28 Gy (0.92–4.0 Gy) and 15 cc of heart in *k*‐DCA plans with an average dose reduction of 0.97 ± 2.2 Gy (0.2–9.0 Gy) when compared to clinical plans.

Normal lung tissue sparing was tracked for V5Gy, V10Gy and MLD because literature suggested these better predict radiation‐induced pneumonitis than V20Gy ^28^, ^29^, ^30^ (Table 2). For V5Gy, V10Gy, V20Gy and MLD, KBPs were able to significantly reduce (all *P* < 0.001) the dose to normal lung when compared to clinical plans. This suggests that in most cases KBPs show reduced normal lung doses and could potentially allow for re‐treatment in future as needed. Clinical plans delivered higher doses to normal lung tissue across all metrics when compared to *k*‐DCA plans, however only V5Gy (*P = *0.006) and V20Gy (*P < *0.001) were statistically significant. This could correlate to a potential lower risk of radiation‐induced pneumonitis via *k*‐DCA plans.

**Table 2:** Evaluation of normal lung doses for all 20 lung stereotactic body radiotherapy (SBRT) validation cases generated using knowledge‐based plannings (KBPs) or k‐dynamic conformal arcs (DCA) routine.

Target	Parameter	Clinical	KBP	*k‐*DCA	Clinical vs KBP	Clinical vs *k*‐DCA	KBP vs *k*‐DCA
Lungs‐PTV	V5Gy (%)	12.0 ± 4.8	11.0 ± 4.4	11.1 ± 4.5	***P < *0.001**	***P = *0.006**	*P = *n. s.
	V10Gy (%)	7.3 ± 3.1	6.7 ± 3.0	6.9 ± 3.0	***P < *0.001**	*P = *n. s.	*P = *n. s.
	V20Gy (%)	2.8 ± 1.3	2.6 ± 1.2	2.5 ± 1.2	***P < *0.001**	***P < *0.001**	***P = *0.005**
	MLD (Gy)	2.48 ± 0.9	2.25 ± 0.9	2.30 ± 0.9	***P < *0.001**	***P = *0.001**	*P = *n. s.

Mean ± SD and *P*‐values were reported. n. s. = not significant. SD = standard deviation. Significant values are highlighted in bold.

**Fig. 3:**
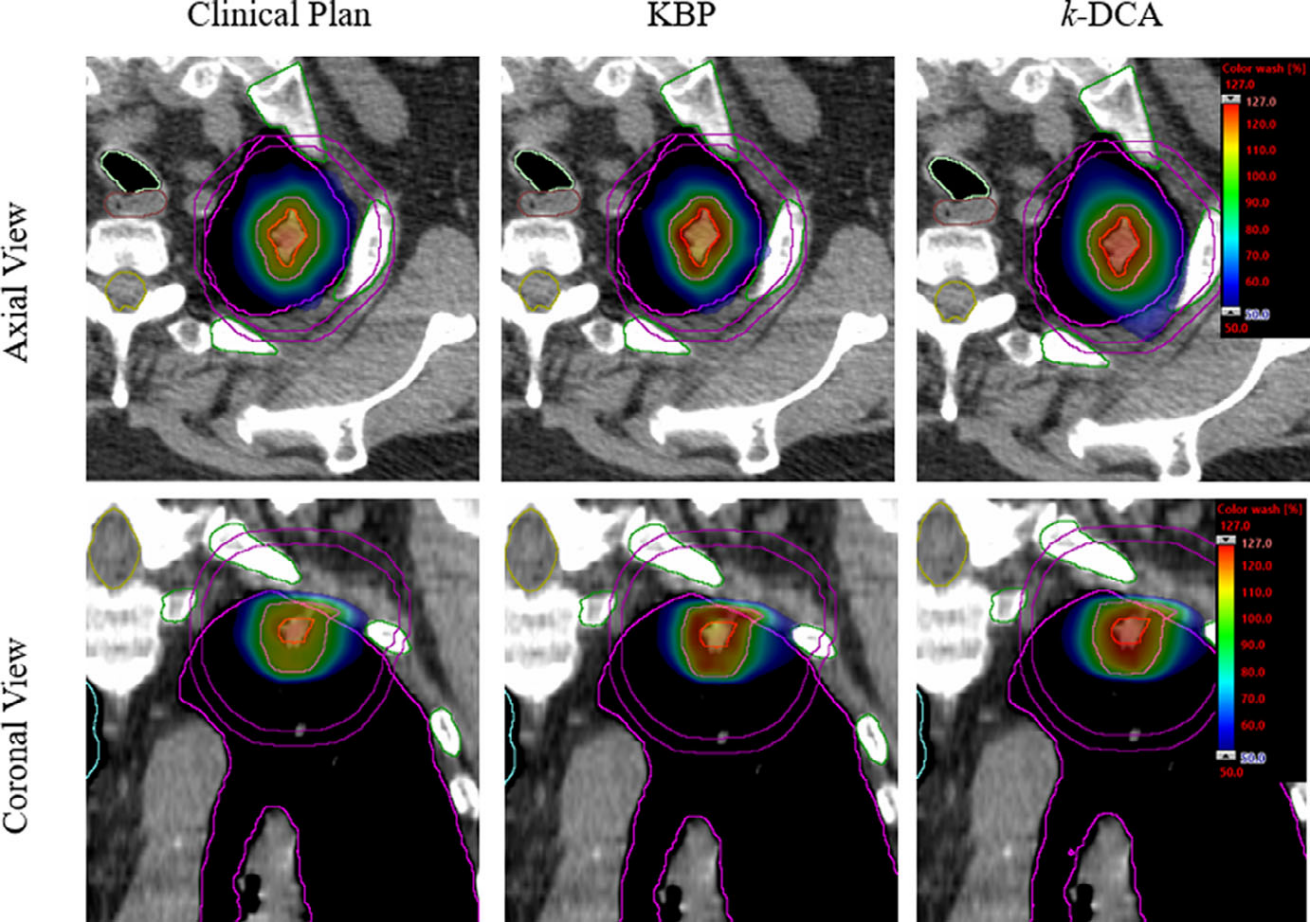
Radiosurgical dose colorwash of the clinical plan, knowledge‐based planning (KBP) and *k*‐dynamic conformal arcs (DCA) plan for a selected validation case. Trachea (light green), esophagus (brown), spinal cord (yellow), and ribs (dark green) are shown. Clinical plan shows a smaller central hotspot than both KBPs and *k*‐DCA plan. 50% isodose colorwash was slightly larger in the *k*‐DCA plan but still clinically acceptable. The largest central hotspot was seen in the *k*‐DCA plan improving dose to GTV.

**Fig. 4:**
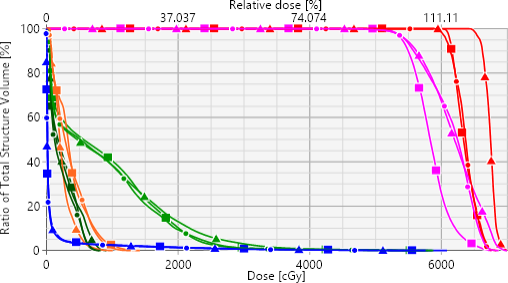
Dose volume histogram for the clinical plan (squares), knowledge‐based planning (circles) and k‐dynamic conformal arcs (DCA) (triangle) plans corresponding to example case presented in Fig. 3. PTV (pink), GTV (red), ribs (green), brachial plexus (orange), trachea (dark green), and lungs‐PTV (blue) are presented. Note for similar OAR sparing, the k‐DCA plan significantly increased GTV dose.

A dose color wash distribution with both the axial and coronal views of an example validation case is shown (Fig. 3). Corresponding dose‐volume histogram is shown in Fig. 4. Select OAR are also shown for reference to the tumor location. Highly conformal radiosurgical dose distribution with a tighter 50% isodose colorwash (blue) can be observed in both clinical and KBPs, however, there was a reduced central hotspot in both plans when compared to the *k‐*DCA plan. This reflects our findings that *k‐*DCA routine was able to increase minimum dose to GTV. This larger central hotspot displayed in the *k*‐DCA was achieved with minimal to no additional costs in OAR dosing. It is shown that the 50% isodose color wash was slightly larger in the *k*‐DCA axial slice but still easily met RTOG‐0618 criteria.

### 3.D Planning efficiency and deliverability

The *k*‐DCA plans were generated using plan geometry identical to previously treated plans in less than 30 min. Table 3 displays the average values of various treatment delivery parameters across all 20 lung SBRT validation cases. The most important result to note here is the significant reduction of total monitor units in the *k*‐DCA plans due to less MLC modulation through the target. On average, *k*‐DCA plans delivered 1133 and 2460 less MU than clinical plans and KBPs, respectively. This correlates to a lower modulation factor, indicating less plan complexity and shorter beam on time. This validates that *k*‐DCA plans are able to create similar or better quality plans than manually generated clinical plans and KBPs with significantly less MLC modulation. Additionally, the in‐house MC second check algorithm reported that the clinical plans (1.8%) and KBPs (2.4%) on averaged showed less agreement with planned TPS dose with respect to k‐DCA plans (1.0%). This indicates the *k*‐DCA planning routine may more accurately deliver treatment. Additionally, the better MC agreement will provide more confidence in treatment delivery accuracy for online/offline adaptive lung SBRT.

**Table 3:** Evaluation of average treatment delivery parameters for all 20‐lung stereotactic body radiotherapy (SBRT) validation cases that were generated using knowledge‐based planning (KBP) or automated *k*‐dynamic conformal arcs (DCA) routine.

Treatment delivery parameter	Clinical	KBP	*k‐*DCA	Clinical vs KBP	Clinical vs *k*‐DCA	KBP vs *k*‐DCA
Total monitor units (MU)	5488 ± 1018	6804 ± 963	4344 ± 687	***P < *0.001**	***P < *0.001**	***P < *0.001**
Modulation factor (MF)	1.02 ± 0.2	1.26 ± 0.2	0.80 ± 0.1	***P < *0.001**	***P < *0.001**	***P < *0.001**
Beam‐on time (min)	3.9 ± 0.7	4.9 ± 0.7	3.1 ± 0.5	***P < *0.001**	***P < *0.001**	***P < *0.001**
MC agreement (%)	±1.76	± 2.40	± 1.03	***P = *0.03**	*P = *n. s.	***P = *0.002**

Mean ± SD and *P*‐values were reported. n. s. = not significant. SD = standard deviation. Significant values are highlighted in bold.

## 4 DISCUSSION

A novel automatic planning routine was developed to generate a non‐coplanar VMAT lung SBRT *k*‐DCA plans in less than 30 min. Both conventional and the *k*‐DCA planning routine generates a similar or better‐quality plan than manually planning. This method reduces inter‐planner variability and lowers the plan complexity when compared to original clinical and conventional knowledge‐based plans. Better target coverage and more DLO sparing was achieved with *k*‐DCA plans because it merges the benefits of both a historical DCA‐based lung SBRT planning approach with a powerful automatic inverse planning engine. Using the 3D DCA‐based dose as a starting point and optimal machine generated DVH estimates, we have shown that a more accurate lung SBRT plan can be generated in a short period of time compared to typical model‐based knowledge‐based planning. Due to less MLC modulation through the target, *k*‐DCA plans could potentially reduce interplay effect and small‐fields dosimetry errors as demonstrated by better agreement with MC calculation. Nonetheless, it remains incumbent on the treating physician to review plans in detail to help ensure accuracy and appropriateness of the objectives of the treatment since there can be intangible goals of the plan that a computer cannot recognize.

Other investigators have created RapidPlan KBPs for lung SBRT and some evaluated plan complexity.^12^, ^16^, ^17^, ^18^ The most recent study by Yu et al. compared clinical VMAT SBRT plans with both the University of California, San Diego’s publicly shared RapidPlan VMAT lung SBRT model and robotic CyberKnife plans.^12^They reported on average an increase of 1025 MU in KBPs when compared to manual clinical plans for a prescription dose of predominately 50 Gy in 5 fractions.^12^ This reflects similar findings in our study but using our automated *k*‐DCA planning routine we were able significantly reduce the total MU (see Table 3). A study by Delaney et al. produced two RapidPlan models intended to treat peripheral lung tumors in either 54 Gy in three treatments or 55 Gy in 5 fractions.^17^ For their 55 Gy evaluation patient group, they report average increase of 222 MU for their 5 × 11 Gy model and 188 MU for their combined prescription model compared to manually generated PO optimized plans.^17^Additionally, they reported an increase of monitor units in their 54 Gy evaluation group of 384 MU and 242 MU for their prescription specific and combined model, respectively. While not as dramatic as the results shown by Yu et al.,^12^ there is an apparent increase of MU when using KBPs which could be accounted for by different choice of planning constraints and model input datasets, similar to one demonstrated by Kubo et al for conventionally fractionated prostate radiotherapy.^31^ With our previous institutional experience, we believe that selecting normal tissue constraints is a critical process in the creation of a KBP model and it will influence the performance of the model as much as the initial input dataset selection. Our previous clinical experience with building a KBP model designed to treat central locations with 50 Gy in 5 fractions scheme also shown increased MU by an average of 261. However, our automated *k*‐DCA planning routine was able to overcome this issue and maintained significantly lower total MU and consequently shorter beam‐on time. Other KBP models were generated but did not report total number of MUs.^16^, ^18^

This automated *k*‐DCA planning routine appears to be the first of its kind and its novelty is further enhanced when validated independently with MC dose calculations. The use of the MC code opens the possibility of using this routinely in the clinic for either online adaptive re‐planning or same/next day offline adaptive re‐planning of lung SBRT treatment. It has previously been shown that 30 Gy in a single fraction can be delivered to the lung lesion in a 15‐min time slot.^32^ Delivering a single fraction treatment subjects the plan to delivery potential errors that could greatly enhanced the interplay effect, so our *k*‐DCA routine could potentially limit this effect by providing less MLC modulation across the target and improve small‐field dosimetry.^33^ Further validation and clinical implementation of this KBP model and automated *k*‐DCA routine for SBRT patient treatment is underway.

## 5 CONCLUSION

A novel lung SBRT KBP model for the treatment of peripherally located early stage NSCLC tumors as defined by RTOG‐0618 was developed and validated. In conjunction with this model, a first of its kind automated *k*‐DCA planning routine was developed to generate high‐quality lung SBRT plans with less complexity. Utilizing this process, a high‐quality lung SBRT treatment plan can be generated in as little as 30 min with less inter‐planner variability, allowing for same day or next day adaptive re‐planning, if desired. Due to less MLC modulation through the target and faster treatment delivery, a *k*‐DCA plan could potentially reduce treatment delivery complexity and intra‐fraction motion errors; improve patient comfort and treatment delivery accuracy.

## AUTHORS CONTRIBUTIONS

DP and JV conceived the project. JV developed and JV and GG validated KBP model, collected and analyzed the data. RM and MR provided clinical expertise and supervision of the paper. JV and DP drafted the manuscript and all co‐authors revised and approved the final manuscript.

## CONFLICT OF INTEREST

None.
